# Supplementary material for: The interactome of intact mitochondria by cross-linking mass spectrometry provides evidence for coexisting respiratory supercomplexes
Source: Mol Cell Proteomics. 2017 Dec 8;17(2):216–32. doi: 10.1074/mcp.RA117.000470 (PMC5795388; doi:10.1074/mcp.RA117.000470)
Supplement: Supplemental Data [file supp_RA117.000470_133922_0_supp_23978_fzffwf.zip › spectra_annotation/mito_DR_spectra_annotation/458-1-14-1-1-1.pdf]

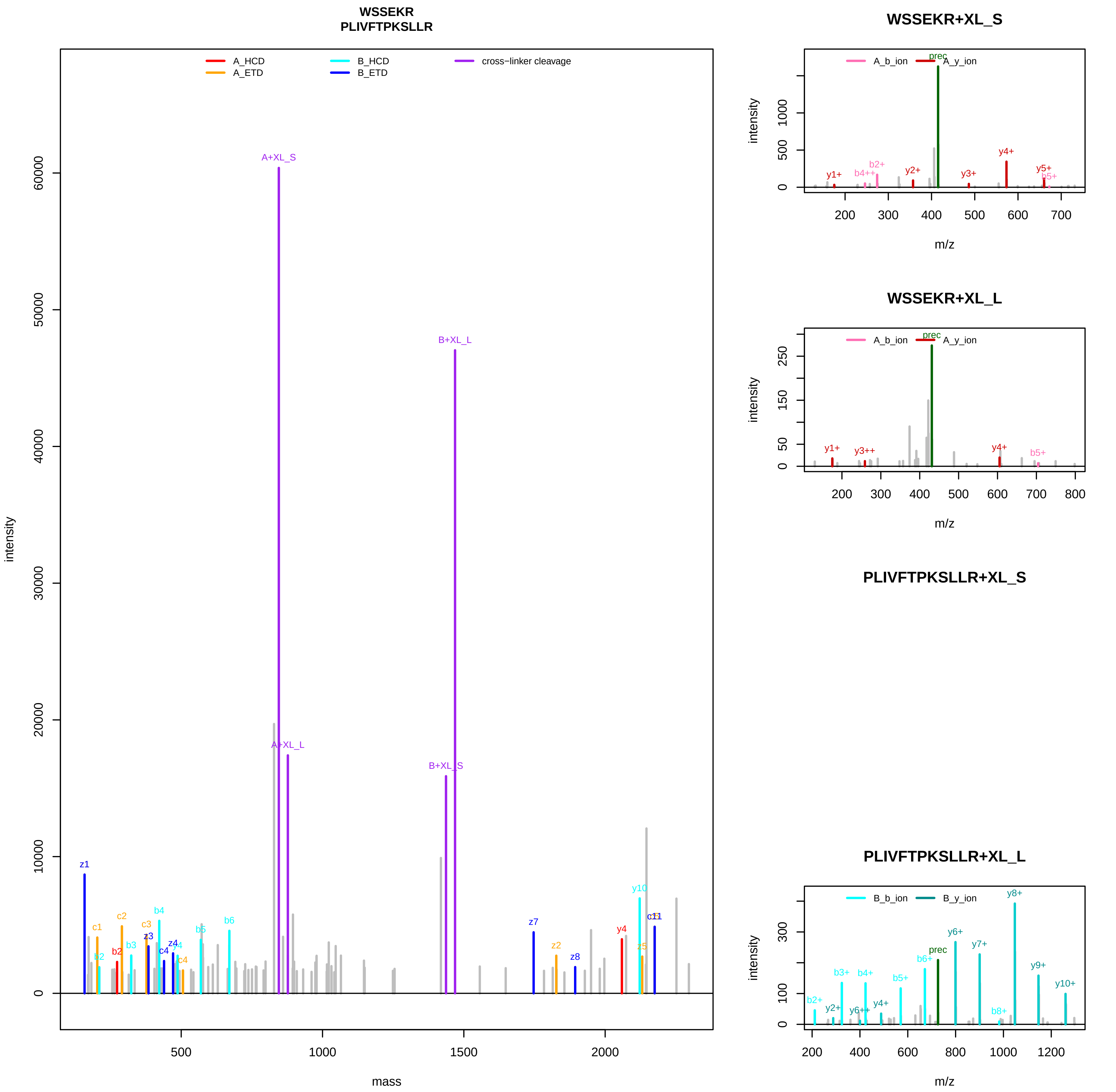
WSSEKR
PLIVFTPKSLLR
A_HCD
A_ETD
B_HCD
B_ETD
cross−linker cleavage
A+XL_S
B+XL_L
A+XL_L
B+XL_S
z1
c1
b2
c2
b2
c3
z3
b3
b4
c4
z4
y4
c4
b5
b6
z7
z2
z8
y4
y10
z5
c11
intensity
mass
0
10000
20000
30000
40000
50000
60000
500
1000
1500
2000
WSSEKR+XL_S
A_b_ion
A_y_ion
prec
y1+
b4++
b2+
y2+
y3+
y4+
y5+
b5+
m/z
WSSEKR+XL_L
y1+
y3++
y4+
b5+
PLIVFTPKSLLR+XL_S
PLIVFTPKSLLR+XL_L
B_b_ion
B_y_ion
b2+
y2+
b3+
y6++
b4+
y4+
b5+
b6+
prec
y6+
y7+
b8+
y8+
y9+
y10+
